# Comparison of Two Diagnostic Assays for Anti-Laminin 332 Mucous Membrane Pemphigoid

**DOI:** 10.3389/fimmu.2021.773720

**Published:** 2021-11-25

**Authors:** Stephanie Goletz, Federica Giurdanella, Maike M. Holtsche, Miranda Nijenhuis, Barbara Horvath, Gilles F. H. Diercks, Detlef Zillikens, Takashi Hashimoto, Enno Schmidt, Hendri H. Pas

**Affiliations:** ^1^ Lübeck Institute for Experimental Dermatology (LIED), University of Lübeck, Lübeck, Germany; ^2^ Center for Blistering Diseases, University of Groningen, Groningen, Netherlands; ^3^ Department of Dermatology, University of Lübeck, Lübeck, Germany; ^4^ Department of Dermatology, Kurume University School of Medicine, Kurume, Japan

**Keywords:** autoantibody, blistering, biochip, cancer, footprint, laminin 332, malignancy, mucous membrane pemphigoid

## Abstract

Anti-laminin 332 mucous membrane pemphigoid (MMP) is an autoimmune blistering disease characterized by predominant mucosal lesions and autoantibodies against laminin 332. The exact diagnosis of anti-laminin 332 MMP is important since nearly 30% of patients develop solid cancers. This study compared two independently developed diagnostic indirect immunofluorescence (IF) tests based on recombinant laminin 332 expressed in HEK239 cells (biochip mosaic assay) and the migration trails of cultured keratinocytes rich in laminin 332 (footprint assay). The sera of 54 anti-laminin 332 MMP, 35 non-anti-laminin 332 MMP, and 30 pemphigus vulgaris patients as well as 20 healthy blood donors were analyzed blindly and independently. Fifty-two of 54 and 54/54 anti-laminin 332 MMP sera were positive in the biochip mosaic and the footprint assay, respectively. In the 35 non-anti-laminin 332 MMP sera, 3 were positive in both tests and 4 others showed weak reactivity in the footprint assay. In conclusion, both assays are easy to perform, highly sensitive, and specific, which will further facilitate the diagnosis of anti-laminin 332 MMP.

## Introduction

Anti-laminin 332 mucous membrane pemphigoid (MMP) is a subepidermal blistering autoimmune disease defined by predominant mucosal lesions and autoantibodies against laminin 332, formerly known as laminin 5 and epilegrin (1–4). Laminin 332 is a heterotrimer consisting of α3, β3, and γ2 chains (5). The protein is part of the dermal–epidermal junction interacting with integrin α6β4, integrin α3β1, BP180 (type XVII collagen), and type VII collagen (5). Anti-laminin 332 MMP comprises 10%–25% of MMP patients (6, 7) and was reported to be associated with malignancies in 25%–30% of patients (3, 4, 8–14). Therefore, a sensitive and specific detection of anti-laminin 332 autoantibodies is of great importance to identify patients at risk of a malignancy and to initiate a tumor search in the anti-laminin 332-reactive MMP patients.

Several in-house methods for the detection of serum anti-laminin 332 IgG have been described including immunoprecipitation, immunoblotting, and ELISA using different cellular sources and recombinant forms of laminin 332 (7, 9, 15–23). Direct comparison of six different methods revealed immunoprecipitation with radiolabeled keratinocyte extracts as the most sensitive technique followed by immunoblotting with extracellular matrix of cultured human keratinocytes (19). Most recently, two indirect immunofluorescence (IF) tests have been described based on the recombinant expression of laminin 332 on the cell surface of a human cell line (biochip mosaic assay) and the migration trails of cultured keratinocytes rich in laminin 332 (keratinocyte footprint assay) (10, 24). Both assays have shown high sensitivities and specificities of 84% and 99.5% (biochip mosaic) and 100% and 100% (footprint assay), respectively (10, 24).

As already suggested by others (25), the aim of the present study was the direct comparison between the two test systems through blind and independent analysis of a high number of well-characterized sera from patients with MMP and pemphigus vulgaris as well as healthy volunteers.

## Materials and Methods

### Human Sera

Sera from patients with anti-laminin 332 MMP used for this study (*n* = 54) were collected at the dermatology departments in Lübeck (Germany), Kurume (Japan), and Groningen (The Netherlands). The criteria for inclusion of patients with anti-laminin 332 MMP were i) clinical phenotype with predominant mucosal lesions, ii) binding of serum IgG along the floor of the artificial blister of salt-split normal human skin by indirect immunofluorescence (IF) microscopy, and/or iii) serum IgG4 against laminin 332 by immunoblotting with extract of extracellular matrix of cultured human keratinocytes, immunoblotting with extract of cultured keratinocytes, or reactivity in an anti-laminin 332 ELISA (11, 19, 24, 26, 27). Additionally, few anti-laminin 332 MMP patients were diagnosed by anti-laminin 332 IgG reactivity by immunoprecipitation with extract of human keratinocytes and/or failure of sera to react with laminin 332-deficient skin and concomitant reactivity with normal human skin by indirect IF (28). Furthermore, sera from 35 non-laminin 332-reactive MMP patients were diagnosed by i) a compatible clinical picture, ii) linear deposits of IgG and/or IgA and/or C3 by direct IF microscopy of a perilesional biopsy, and/or iii) reactivity with BP180 by ELISA (Euroimmun, Lübeck, Germany), and/or iv) binding of serum IgG along the roof of the artificial blister of salt-split normal human skin by indirect IF microscopy, and/or v) immunoblotting with conditioned medium of cultured human keratinocytes or extracts of cultured keratinocytes (6, 26, 27). Sera from patients with pemphigus vulgaris (PV, *n* = 30) identified by i) a compatible clinical picture and ii) positive direct IF microscopy of a perilesional biopsy and/or iii) serum IgG against desmoglein 3 by ELISA (Euroimmun) (29) as well as sera from healthy blood donors (HBD, *n* = 20) served as additional controls. The study was approved by the ethics committee of the University of Lübeck (12–178) following the Declaration of Helsinki. Sera were stored at −20°C or −80°C until used.

### Indirect Immunofluorescence Assay Using Recombinant Laminin 332 (Biochip Mosaic)

**Figure 1:**
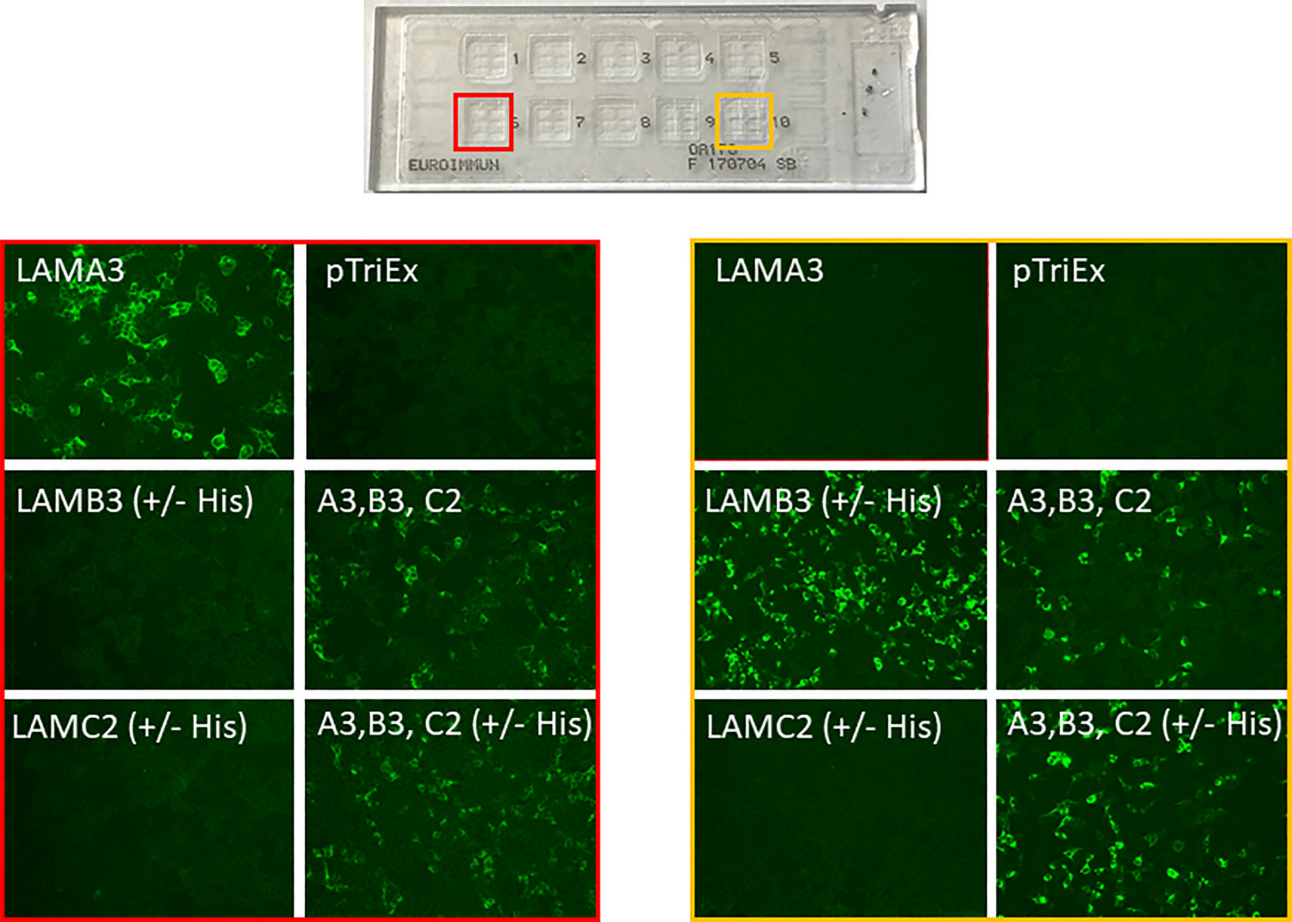
Representative pictures of the indirect immunofluorescence biochip mosaic assay using membrane-bound recombinant laminin α3 (LAMA3), β3 (LAMB3), γ2 (LAMC2), the α3β3γ2 heterotrimer (A3, B3, C2) with and without His-tag, and empty plasmid (pTriEx) expressed in HEK293 cells. Sera from patients with anti-laminin 332 MMP with anti-laminin α3 reactivity (red box) and anti-laminin β3 reactivity (yellow box).

All sera were subjected to the indirect IF biochip mosaic with six different substrates comprising HEK293 cells transfected with plasmids for i) *LAMA3*, ii) *LAMB3*, iii) *LAMC2* (encoding for the α3, β3, and γ2 chains, respectively), iv) all three plasmids encoding for the heterotrimer, v) all three plasmids encoding for the heterotrimer and a His-tag, and vi) the empty plasmid, as described recently (Euroimmun) (10) (**Figure 1**). All sera were applied in a 1:10 dilution in PBS supplemented with 0.2% Tween-20, and after washing, bound autoantibodies were detected by anti-human IgG+IgG4-FITC (Euroimmun). Pictures were taken using a Biorevo Keyence BZ-9000 fluorescence microscope (Keyence Deutschland GmbH, Neu-Isenburg, Germany).

### Indirect Immunofluorescence Assay Using Native Laminin 332 (Footprint Assay)

The keratinocyte footprint assay was prepared and performed as recently described (24). In brief, primary normal human keratinocytes were grown on glass coverslips for 3 days to a confluency about 30%, air-dried, and stored at −20°C until used. The migration trails of the cells left on the coverslips are rich in laminin 332 but do not contain other autoantigens of pemphigoid diseases such as BP180, p200, and type VII collagen. Sera were diluted 1:10 in PBS/ovalbumin. For the detection of bound anti-laminin 332 antibodies, a DyLight488-labeled goat anti-human IgG antibody (Thermo Fisher Scientific, Waltham, MA, USA) was used.

### Indirect Immunofluorescence

All sera (diluted 1:10 in PBS) were analyzed by indirect IF microcopy on 6 µm cryosections of human salt-split skin using a FITC-conjugated monoclonal anti-human IgG detection antibody (1:50; Sigma Aldrich, Munich, Germany).

### Immunoblotting With Extracellular Matrix of Cultured Human Keratinocytes

Preparation of extract of extracellular matrix of cultured keratinocytes, sodium dodecyl sulfate polyacrylamide gel electrophoresis (SDS-PAGE), transfer to nitrocellulose, and immunoblotting were performed as previously described (10, 30). After blocking, nitrocellulose membranes were incubated with human sera (1:50), rabbit IgG against the α3 chain of laminin 332 (1:10,000, Sigma Aldrich, Munich, Germany), monoclonal mouse IgG against the β3 and γ2 chains (clone A-6, 1:100,000; clone E-6, 1:10,000, respectively; both Santa Cruz Biotechnology, Dallas, TX, USA), diluted in TBS with 0.5% Tween-20 (TBS-T) containing 5% skimmed milk and 1% BSA. After washing with TBS-T twice for 12 min, the secondary antibodies, horseradish peroxidase (HRP)-conjugated monoclonal mouse anti-human IgG4 antibody (1:10,000, Southern Biotech, Birmingham, AL, USA), polyclonal rabbit anti-mouse IgG antibody (1:100,000, DAKO, Glostrup, Denmark), and polyclonal goat anti-rabbit antibody (1:10,000, DAKO) were used. After 1 h of incubation, an additional washing step with TBS-T was performed. The proteins were visualized using ECL prime detection systems (GE Healthcare Europe, Freiburg, Germany).

## Results

### Characteristics of Anti-Laminin 332 MMP Patients

We included 54 anti-laminin 332 MMP patients (21 females, 33 males) in our study (**Table S1**). Sixteen of these sera were already described by Giurdanella et al. (24). Most of the other sera were used for the establishment of the indirect IF test using recombinant laminin 332 (10). Direct IF microscopy results were available from 24 patients and were positive in 23 cases. Dermal binding of IgG by indirect IF on salt-split skin was found in 50 (92.6%) patients, and IgG reactivity against laminin 332 was present in 46 (85.2%) patients by immunoblotting with extract of extracellular matrix of cultured human keratinocytes, 6 of 6 patients by laminin 332 ELISA, 5 of 5 patients by indirect IF on normal human but not on laminin 332-deficient skin, and 1 of 2 patients by immunoprecipitation with extract of human keratinocytes. From 25 (46.3%) patients, additional clinical information was available. Eleven (44%) of the 25 anti-laminin 332 MMP patients with clinical data had a malignancy at the time of diagnosis.

### Detection of Autoantibodies Against Laminin 332 Using Recombinant Laminin 332 (Biochip Mosaic)

Sera of patients with anti-laminin 332 MMP (*n* = 54), other MMP (*n* = 35), PV (*n* = 30), and HBD (*n* = 20) were analyzed by the laminin 332 biochip assay (**Figure 1**). Fifty-two (96.3%) of the anti-laminin 332 MMP sera showed reactivity with the laminin 332 heterotrimer with or without His-tag (**Table 1**). Of all the 54 sera, 35 (64.8%) reacted with the α3 chain, 21 (38.9%) with the β3 chain, and 6 (11.1%) with the γ2 chain (**Table 1**). One serum (1.9%) reacted only with the heterotrimer (**Table 1** **)**. In the group of the original 35 non-anti-laminin 332 MMP sera, 7 sera were reactive with laminin 332 by foot print assay as described below, and 3 of these 7 sera also reacted with laminin 332 in the biochip mosaic (**Tables 1**, **2**). No positive reactivity was seen with the PV and HBD sera (**Table 2**).

**Table 1:** Reactivity of the anti-laminin 332 mucous membrane pemphigoid sera with the different laminin 332 chains by biochip mosaic assay.

	Biochip assay					
	α3	β3	γ2	α3β3γ2 heterotrimer	His-α3β3γ2 heterotrimer	pTriEx (negative control with empty plasmid)
**Anti-laminin 332 MMP patients**	35/54	21/54	6/54	52/54	52/54,	0/54
	64.8%	38.9%	11.1%	96.3%	96.3%	0%
	52/54, 96.3%					
**Additional laminin 332-positive MMP sera (footprint positive)**	2/7	1/7	0/7	3/7	3/7	0/7
	28.6%	14.3%	0%	42.9%	42.9%	0%
	3/7					

**Table 2:** Comparison of anti-laminin 332 reactivity using the biochip and the footprint assay.

	Biochip assay	Footprint assay
Anti-laminin 332 mucous membrane pemphigoid	52/54	54/54
	96.3%	100%
Non-anti-laminin 332 mucous membrane pemphigoid	3/35	7/35
	8.6%	20%
Pemphigus vulgaris	0/30	0/30
	0%	0%
Healthy blood donors	0/20	0/20
	0%	0%
Sensitivity^a^	55/61	61/61
	90.2%	100%
Specificity	100%	100%

a Sixty-one sera; in seven sera of patients originally classified as non-anti-laminin 332 mucous membrane pemphigoid, an additional anti-laminin 332 IgG was detected in this study.

### Detection of Autoantibodies Using Native Laminin 332 (Footprint Assay)

**Figure 2:**
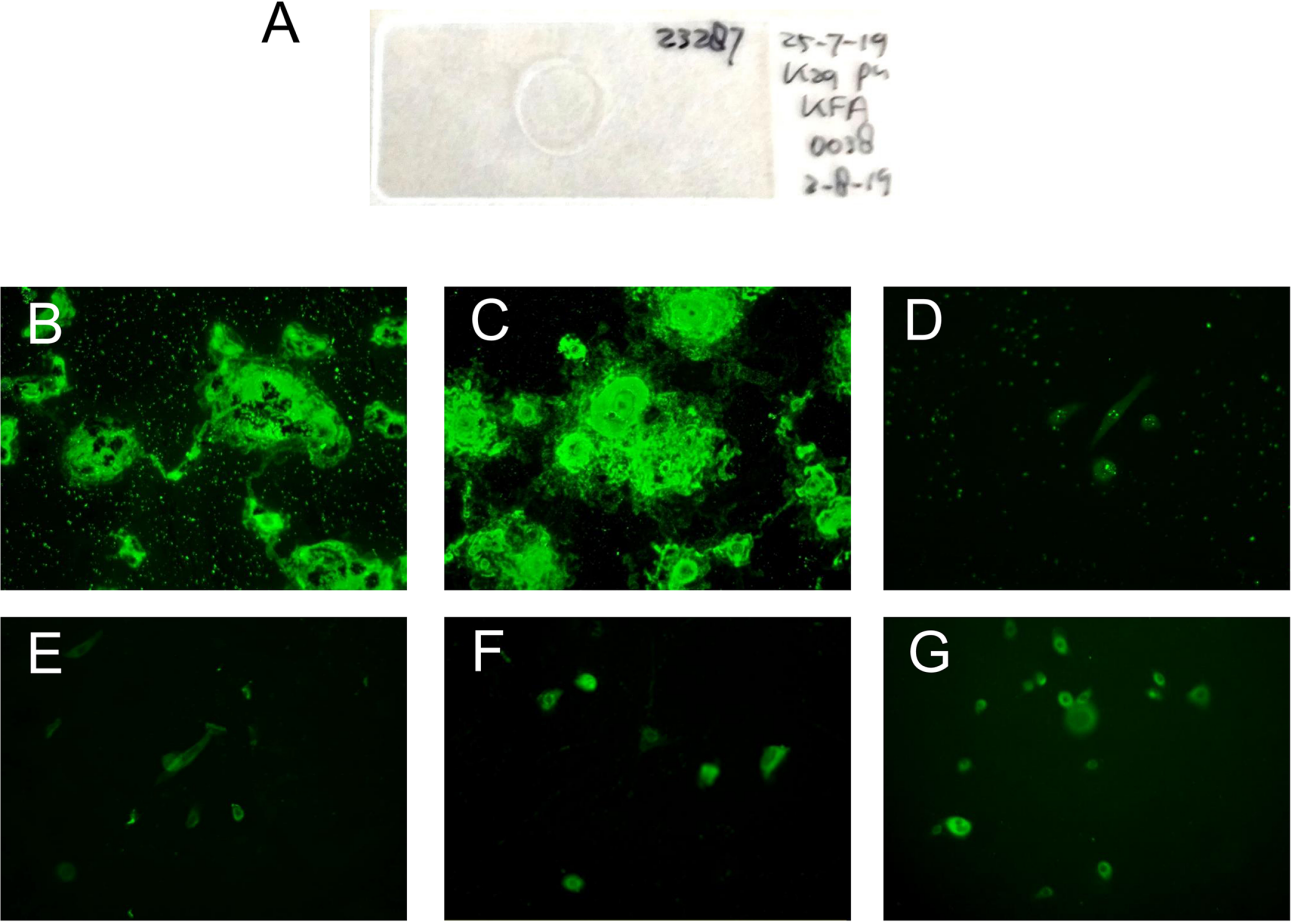
Representative pictures of the indirect immunofluorescence-based keratinocyte footprint assay. **(A)** Example of an incubation slide with normal human keratinocytes grown on a glass coverslip. **(B, C)** MMP patient serum IgG binds to deposited laminin 332 on air-dried coverslips. **(D)** Serum of a healthy blood donor. **(E)** Serum of a bullous pemphigoid patient. **(F)** Serum of an epidermolysis bullosa acquisita patient. **(G)** Serum of an anti-p200 pemphigoid patient.

In the keratinocyte footprint assay (**Figure 2**), all the tested 54 anti-laminin 332 MMP sera (100%) showed reactivity (**Table 2**). As describe above, seven sera from patients originally classified as MMP without laminin 332 reactivity were reactive to the footprints (**Table 2**). All PV and HBD sera were negative (**Table 2**). To demonstrate the specificity of the laminin 332-specific staining, pictures of additional controls (anti-p200 pemphigoid, BP, and EBA) are shown (**Figure 2**).

### Detection of Laminin 332 Autoantibodies by Immunoblotting

Sera of all patients with anti-laminin 332 MMP (*n* = 54) were analyzed by immunoblotting with extracellular matrix of cultured human keratinocytes. Forty-six of 54 sera (85.2%) showed IgG4 reactivity with at least one chain in the former assay (**Table S1**).

## Discussion

Patients with anti-laminin 332 MMP cannot be differentiated from other variants of MMP based on clinical appearance. However, identification of MMP patients with laminin 332-specific antibodies is essential since 25%–30% of these patients might have a malignancy (3, 4, 8–14). The introduction of the laminin 332-specific biochip mosaic in 2019 provided us with widely available standardized test system for the detection of anti-laminin 332 serum autoantibodies. In an initial study, this test showed a sensitivity of 84% and a specificity of 99.8% by investigating sera from anti-laminin 332 MMP patients and controls (10). In the same year, another indirect IF test for the detection of laminin 332-specific serum IgG based on the laminin 332-rich migration trails of cultured keratinocytes, so-called footprint assay, was established (24). The latter assay showed a sensitivity of 100% and a specificity of 97.1% (24). The aim of the present study was to compare the performance of these two recently established laminin 332-specific indirect IF tests in a blind and independent approach.

Both assays were easy to perform and revealed 100% specificity with very high sensitivities, i.e., 52 of 54 (96.2%) and all the 54 (100%) anti-laminin 332 MMP sera showed positive reactivity in the biochip assay and the footprint assay, respectively. Interestingly, seven additional MMP sera, which had previously been considered to react with BP180, showed positive reactivity in the footprint assay. Among the seven sera, three sera also show positive reactivity with the laminin heterotrimers (two to α3 subunit and one to β3 subunit) in biochip mosaic assay.

These results reflect the high sensitivities of the two assays compared with the sensitivities of the previously applied tests in this cohort that had excluded anti-laminin 332 antibodies in the MMP sera. It also shows that anti-laminin 332 antibodies, albeit at a low level, can also be present in patients with anti-BP180 MMP. Such patients have been described before (31, 32). At present, it is unknown whether these additional anti-laminin 332 antibodies influence the clinical phenotype. Our data suggest that testing for anti-laminin 332 reactivity may also be valuable in MMP patients with anti-BP180 reactivity. This view is supported by the observation of Bernard and coworkers that mucous membrane pemphigoid patients showed an association of anti-BP230 and anti-laminin 332 autoantibodies, both measured by ELISA (33). In line with this, the recent European guidelines on diagnosis and management of MMP recommended testing for laminin 332-specific autoantibodies also in cases with negative indirect IF in salt-split skin (3, 4).

The slightly higher sensitivity of the footprint assay may be explained by the specific pattern recognition, which helps to identify also very weak signals. The background staining can be easily differentiated from the specific pattern. In the biochip, untransfected cells that do not express laminin 332 serve as internal controls; however, they may show some autofluorescence. These observations also reflect the reported higher sensitivity of the footprint assay compared with the biochip (10, 24).

Furthermore, several other assays for the detection of serum antibodies against laminin 332 have been established in specialized laboratories including immunoprecipitation using cultured keratinocytes, ELISA, and IB applying purified or cell-derived laminin 332 or recombinant forms of the laminin α3 chain with varying sensitivities (2%–90%, dependent on the cohort) and specificities from 82% to 100% (7, 9, 15–18, 20–23). However, none of these tests was commercially available.

The differences observed in **Table S1** between the immunoblot applying extract of keratinocytes and the immunoblot using extracellular matrix of cultured keratinocytes in the first 16 sera may be due to the different extracts used in our laboratories, as well as to the detection antibody directed against total IgG and IgG4, respectively.

The high association of malignant solid tumors with anti-laminin 332 MMP has initially been noticed by Egan et al. (8), which had been subsequently confirmed by five other studies showing malignancies in 21 (30%) of 70 patients (9–13). In contrast, Bernard and coworkers detected anti-laminin 332 IgG by a laminin 332-specific ELISA in the sera of 31 of 154 MMP patients including only 2 (6%) patients with malignant tumors (33). When all 19 ELISA-positive sera from one study center were reanalyzed by the laminin 332-specific biochip mosaic, 4 patients were reactive, 1 of which had a malignancy (unpublished data; with kind permission of Frank Antonicelli, Reims, France).

In the present study, we found an associated malignancy in 11 of our 25 (44%) anti-laminin 332 MMP patients where clinical data in addition to the clinical phenotype could be retrieved from the patient charts. Most of these cases were already included in our recent studies for the establishment of the two laminin 332-specifc test systems (10, 24) (**Table S1**). The retrospective search for malignant tumors in the records of our patients may account for the higher percentage of malignancies in our cohorts compared with the 25%–30% of patients in previous studies (8–13). Of interest, of the seven sera that were originally included as from non-laminin 332-reactive MMP patients, but showed laminin 332 reactivity in the footprint assay, two patients, of which one was also positive on the biochip, revealed malignancies supporting the high sensitivity of both assays.

In summary, the two recently established indirect IF assays for the detection of serum anti-laminin 332 IgG are highly sensitive and specific and easy to perform. While the footprint assay is slightly more sensitive, the biochip mosaic is highly standardized and widely available. Both assays will further facilitate the serological diagnosis of anti-laminin 332 MMP allowing initiating a tumor screening in MMP patients with anti-laminin 332 reactivity.

## Data Availability Statement

The original contributions presented in the study are included in the article/**Supplementary Material**. Further inquiries can be directed to the corresponding author.

## Ethics Statement

The studies involving human participants from Lübeck were reviewed and approved by the ethics committee of the University of Lübeck (12–178). Written informed consent for participation from Groningen was not required for this study in accordance with the national legislation and the institutional requirements.

## Author Contributions

SG contributed to the performance of the experiments and the writing of the manuscript. FG, MN, and GD contributed to the performance of the experiments and the revision of the manuscript. SG, ES, and HP contributed to the planning of the project. MH, BH, DZ, and TH contributed to the revision of the manuscript. HP and ES contributed to the writing of the manuscript. All authors contributed to the article and approved the submitted version.

## Funding

This work was supported by structural funding from the Schleswig-Holstein Excellence Cluster *Precision Medicine in Chronic Inflammation* (DFG EXC 2167/1) and the CRU 303 *Pemphigoid Diseases* (to DZ and ES) Biochip mosaics were provided free of charge by Euroimmun, Lübeck, Germany.

## Conflict of Interest

DZ and ES have obtained grants from Euroimmun, Lübeck, for research and development projects.

The remaining authors declare that the research was conducted in the absence of any commercial or financial relationships that could be construed as a potential conflict of interest.

## Publisher’s Note

All claims expressed in this article are solely those of the authors and do not necessarily represent those of their affiliated organizations, or those of the publisher, the editors and the reviewers. Any product that may be evaluated in this article, or claim that may be made by its manufacturer, is not guaranteed or endorsed by the publisher.
