# Supplementary material for: Comparison of Two Diagnostic Assays for Anti-Laminin 332 Mucous Membrane Pemphigoid
Source: Front Immunol. 2021 Nov 25;12:773720. doi: 10.3389/fimmu.2021.773720 (PMC8657402; doi:10.3389/fimmu.2021.773720)
Supplement: Supplementary file 1 [file Table_1.docx]

**Table S1**

**Patients characteristics**

|  | **Characteristics** | | | | | | | | | | **This study** | | | |
| --- | --- | --- | --- | --- | --- | --- | --- | --- | --- | --- | --- | --- | --- | --- |
|  | **Gender (M/F)** | **Age** | **DIF** | **SSS**  **(dermal binding)** | **IB laminin 332 (IgG)** | **IB ECM (IgG4)** | **ELISA Ln-332** | **IIF Col7 KO Skin** | **IIF Ln-332 KO Skin** | **Cancer** | **SSS (dermal binding)** | **Foot-print** | **Biochip** | **IB ECM (IgG4)** |
| **No.** |  | | | | | | | | | | | | | |
| 1^a,b ,c^ | F | 64 | DIF+ | + | α3*^1^ | np | + | np | np | no | + | + | + | α3 |
| 2^b, c^ | F | 65 | DIF+ | + | α3 | np | + | np | np | no | + | + | + | α3 |
| 3^b, c^ | M | 56 | DIF+ | IgG dubious | α3 | np | + | np | np | yes | + | + | - | BP180 |
| 4^b, c^ | M | 27 | DIF+ | + | α3 | np | + | np | np | no | + | + | + | α3 |
| 5^b, c^ | M | 25 | DIF+ | + | α3 | np | + | np | np | no | + | + | + | - |
| 6^b, c^ | M | 70 | DIF+ | + | α3*^2^ | np | np | np | np | yes | + | + | + | α3 |
| 7^b,c^ | F | 42 | DIF+ | + | α3 | np | + | np | np | no | + | + | + | α3 |
| 8^c^ | F | 86 | DIF+ | + | γ2 | np | np | + | - | no | + | + | + | - |
| 9^c^ | M | 65 | DIF+ | + | α3 | np | np | np | np | no | + | + | + | α3 |
| 10^c, d^ | F | 52 | DIF+ | + | - | np | np | + | - | no | + | + | + | - |
| 11^c^ | M | 77 | np | + | α3, β3 | np | np | + | - | na | + | + | + | β3 |
| 12^c^ | F | 59 | - | + | α3, β3 | np | np | + | - | no | + | + | + | β3 |
| 13^c^ | M | 54 | DIF+ | IgG+, IgA | - | np | np | + | - | yes | + | + | + | - |
| 14^c^ | M | 63 | DIF+ | + | α3 | np | np | np | np | na | + | + | + | α3 |
| 15^c^ | M | 81 | np | + | α3 | np | np | np | np | na | + | + | + | α3 |
| 16^c^ | M | 82 | DIF+ | + | α3, β3, γ2 | np | np | np | np | no | + | + | + | BP180 |
| 17^e^ | M | 86 | n.a. | - | np | α3 | np | np | np | na | - | + | + | - |
| 18^e^ | F | 43 | DIF+ | - | np | α3 | np | np | np | na | + | + | + | α3 |
| 19^e^ | F | na | na | + | np | β3 | np | np | np | no | + | + | + | β3 |
| 20^e^ | M | 83 | na | + | np | β3 | np | np | np | na | + | + | + | β3 |
| 21^e^ | M | 91 | na | + | np | β3 | np | np | np | na | + | + | + | β3 |
| 22^e^ | M | 76 | na | + | np | γ2 | np | np | np | na | + | + | + | γ2 |
| 23^e^ | F | 70 | na | + | np | β3 | np | np | np | na | + | + | + | β3, γ2 |
| 24^e^ | F | 52 | na | + | np | α3 | np | np | np | na | + | + | + | α3 |
| 25^e^ | F | 73 | na | + | np | α3 | np | np | np | na | + | + | + | α3 |
| 26^e^ | M | 73 | na | + | np | α3 | np | np | np | na | + | + | + | α3 |
| 27^e^ | M | 73 | na | + | np | α3 | np | np | np | yes | + | + | + | α3 |
| 28^e^ | F | 40 | na | + | np | α3, β3 | np | np | np | na | + | + | + | α3, β3 |
| 29^e^ | F | 41 | na | + | np | α3, β3 | np | np | np | na | + | + | + | α3, β3 |
| 30^e^ | F | 45 | na | + | np | α3 | np | np | np | yes | + | + | + | α3 |
| 31 | M | 66 | na | - | np | γ2 | np | np | np | na | - | + | + | γ2 |
| 32 | F | 68 | na | + | np | β3 | np | np | np | na | + | + | + | β3 |
| 33^e^ | M | 44 | na | + | np | α3 | np | np | np | na | + | + | + | α3 |
| 34^e^ | M | 71 | na | + | np | α3 | np | np | np | na | + | + | + | α3 |
| 35^e^ | M | 75 | DIF+ | + | np | α3,β3 | np | np | np | yes | + | + | + | α3, β3 |
| 36^e^ | F | 88 | na | + | np | α3 | np | np | np | na | + | + | + | α3 |
| 37^e^ | F | 56 | DIF+ | + | np | α3 | np | np | np | yes | + | + | + | α3 |
| 38^e^ | M | 64 | na | + | np | α3 | np | np | np | na | + | + | + | α3 |
| 39^e^ | M | 53 | DIF+ | + | np | α3,β3 | np | np | np | yes | + | + | + | α3, β3 |
| 40^e^ | F | 89 | na | + | np | α3,β3 | np | np | np | na | + | + | + | α3, β3 |
| 41^e^ | M | 74 | na | - | np | α3 | np | np | np | na | - | +  (weak) | - | α3 |
| 42 | F | 67 | na | + | np | α3 | np | np | np | na | + | + | + | α3 |
| 43 | M | 59 | DIF+ | + | np | β3 | np | np | np | no | + | + | + | β3 |
| 44^e^ | M | 47 | na | + | np | α3 | np | np | np | no | + | + | + | α3 |
| 45 | F | 79 | DIF+ | + | np | α3 | np | np | np | yes | + | + | + | α3 |
| 46 | M | 61 | DIF+ | + | np | α3, β3 | np | np | np | no | + | + | + | α3, β3 |
| 47 | M | 78 | na | + | np | α3 | np | np | np | na | + | + | + | α3 |
| 48 | M | 79 | DIF+ | + | np | α3 | np | np | np | na | - | + | + | α3 |
| 49^e^ | M | 69 | DIF+ | + | np | α3 | np | np | np | yes | + | + | + | α3 |
| 50^e^ | M | 77 | na | + | np | α3 | np | np | np | na | + | + | + | α3 |
| 51^e^ | M | 36 | na | + | np | α3 | np | np | np | na | + | + | + | α3 |
| 52^e^ | M | 77 | na | + | np | α3 | np | np | np | na | + | + | + | α3 |
| 53^e^ | F | 46 | na | + | np | α3, β3 | np | np | np | na | + | + | + | α3, β3 |
| 54 | M | 61 | DIF+ | + | γ2 | np | np | np | np | yes | + | + | + | - |

F, female; M, male; DIF, direct immunofluorescence; IIF, indirect immunofluorescence; SSS, IIF of salt-split skin; IB, immunoblot; ECM, extracellular matrix. ^*1^, immunoprecipitation negative ^*2^, immunoprecipitation positive (performed with radiolabeled human keratinocyte extract); +, positive; -, negative; na, not available; np, not performed; KO skin, knock-out skin. ^a^, Jonkman et al. (36) ; ^b^, Terra et al.(13), ^c^, Giurdanella et al. (25); ^d^, van den Bos et al. (37); ^e^, Goletz et al. (12), *, genital lesions
